# Metrological Characterization of a High-Temperature Hybrid Sensor Using Thermal Radiation and Calibrated Sapphire Fiber Bragg Grating for Process Monitoring in Harsh Environments

**DOI:** 10.3390/s22031034

**Published:** 2022-01-28

**Authors:** René Eisermann, Stephan Krenek, Tobias Habisreuther, Petra Ederer, Sigurd Simonsen, Helge Mathisen, Tino Elsmann, Frank Edler, Daniel Schmid, Adrian Lorenz, Åge Andreas Falnes Olsen

**Affiliations:** 1 Physikalisch-Technische Bundesanstalt (PTB), Abbestraße 2-12, 10587 Berlin, Germany; rene.eisermann@ptb.de (R.E.); petra.ederer@ptb.de (P.E.); frank.edler@ptb.de (F.E.); daniel.schmid@ptb.de (D.S.); 2 Leibniz-Institute of Photonic Technology, Albert-Einstein-Str. 9, 07745 Jena, Germany; tobias.habisreuther@leibniz-ipht.de (T.H.); tino.elsmann@leibniz-ipht.de (T.E.); adrian.lorenz@leibniz-ipht.de (A.L.); 3 Elkem ASA Technology, P.O. Box 8040 Vaagsbygd, 4675 Kristiansand, Norway; 4 Norwegian Metrology Service, P.O. Box 170, 2027 Kjeller, Norway; aao@justervesenet.no

**Keywords:** high-temperature sensor, fiber Bragg grating (FBG), sapphire fiber, harsh environment

## Abstract

Fiber Bragg gratings inscribed in single crystalline multimode sapphire fibers (S-FBG) are suitable for monitoring applications in harsh environments up to 1900 °C. Despite many approaches to optimize the S-FBG sensor, a metrological investigation of the achievable temperature uncertainties is still missing. In this paper, we developed a hybrid optical temperature sensor using S-FBG and thermal radiation signals. In addition, the sensor also includes a thermocouple for reference and process control during a field test. We analyzed the influence of the thermal gradient and hotspot position along the sensor for all three detection methods using an industrial draw tower and fixed point cells. Moreover, the signal processing of the reflected S-FBG spectrum was investigated and enhanced to determine the reachable measurement repeatability and uncertainty. For that purpose, we developed an analytical expression for the long-wavelength edge of the peak. Our findings show a higher stability against mechanical-caused mode variations for this method to measure the wavelength shift compared to established methods. Additionally, our approach offers a high robustness against aging effects caused by high-temperature processes (above 1700 °C) or harsh environments. Using temperature-fixed points, directly traceable to the International System of Units, we calibrated the S-FBG and thermocouple of the hybrid sensor, including the corresponding uncertainty budgets. Within the scope of an over 3-weeks-long field trial, 25 production cycles of an industrial silicon manufacturing process with temperatures up to 1600 °C were monitored with over 100,000 single measurements. The absolute calibrated thermocouple ($U_{k=2}\approx 1\;K\ldots 4\;K$) and S-FBG ($U_{k=2}\approx 10\;K\ldots 14\;K$) measurements agreed within their combined uncertainty. We also discuss possible strategies to significantly reduce the uncertainty of the S-FBG calibration. A follow-up measurement of the sensor after the long-term operation at high temperatures and the transport of the measuring system together with the sensor resulted in a change of less than 0.5 K. Thus, both the presented hybrid sensor and the measuring principle are very robust for applications in harsh environments.

## 1. Introduction

Decades of research has established fiber optics-based temperature sensors as viable tools in harsh environments and over long distances [1,2,3]. Compared to electrical or electro-mechanical sensors, fiber sensors offer important benefits such as electrically passive operation, electromagnetic interference immunity, high sensitivity, and multiplexing capabilities [1,2]. They also provide a platform for a rich variety of sensing techniques. The fiber may be used as a light guide for signals generated at or near the fiber tip, such as blackbody or phosphor radiation. It may also be used as the sensor and signal guide, for instance, with Brillouin scattering-based thermometry or inscribed fiber Bragg gratings (FBG). FBGs as thermal sensors probe the change in optical pathlength as a function of thermal expansion and thermally induced change in the refractive index: by illuminating the FBG with a light source and detecting the reflection spectrum, it is possible to accurately track how the optical pathlength changes. Implementations of fiber optical sensors can be found in numerous applications, e.g., condition monitoring of power grid lines [4], gas turbines [5], or monitoring of composite pressure vessels [6].

Silica fibers have been proven to provide reliable measurement data between −260 °C and 600 °C [7,8], but, for higher temperatures, their application has proven challenging. It has been shown that optical fibers show viscoelastic behavior under load starting at approx. 700 °C [9]. The upper temperature limit is around 1000 °C [10], where fused silica starts to devitrify, rendering the fiber unusable for optical applications. However, in vivo sensing in harsh environments, such as gas turbines [5] and furnaces [11], gasifiers [12], or testing the fire resistance of fuel tank materials [13], can easily reach significantly higher temperatures and pressures. Sapphire fibers are a promising alternative as they remain optically and mechanically stable at very high temperatures. It is possible, albeit challenging, to inscribe FBGs in sapphire fibers [14,15,16,17]. Habisreuther et al. [11] previously reported using a sapphire FBG (S-FBG) sensor up to 1500 °C in an induction furnace. The sensor offered a temperature resolution of ±2 K at a monitoring rate of 20 Hz [11], but a quantification of the measurement uncertainty is still missing, as can be seen in [11,17]. The FBG was found to even withstand exposure to 1900 °C [11]. Recent reports also show that single-mode sapphire fibers may successfully be used for the purpose, although their small diameter usually requires some form of cladding as mechanical support, which tends to limit the operational temperature range [18,19]. Hence, for the highest temperatures, one would typically be required to use a thicker sapphire fiber to ensure sufficient mechanical robustness. Unfortunately, a thicker fiber is optically harder to work with. With a typical operating wavelength around 1550 nm, a 100 µm diameter sapphire fiber will support multiple transmission modes. Since each mode will have different transmission characteristics, this leads to a wide and asymmetric peak in the reflection spectrum of a S-FBG. Moreover, the crystalline properties of sapphire enhanced the multimodal broadening of the grating reflection peak. Being a single crystal, the cross section is not perfectly circular, and this causes mode mixing effects and signal loss, especially for higher modes along the entire length of the fiber.

The combined effect smears the reflection peak in an asymmetric manner, with the consequence that data processing is more challenging [17]. However, since the shape of the irregular peaks results from the optical and geometrical properties of the fiber, this pattern is stable and can be used for sensing applications.

One way to improve the peak shape is to change the optical properties of the fiber by applying a cladding, which is able to reduce the number of modes. However, this can be technically very challenging and, depending on the selected material, can limit the upper operating temperature [20].

In addition to the S-FBG, it is possible to use the sapphire fiber itself as a blackbody thermometer. Dils [21] first reported on a device using a sputtered iridium tip as a blackbody, whose thermal radiation follows Planck’s law and was transmitted through the fiber to a detection system at the low temperature end. It is also possible to use the fiber as a guide for thermal radiation from an external cavity, but this will complicate calibration and traceability. However, it opens up the possibility to utilize two fundamentally different physical processes to measure temperature in the same sensor: the thermal radiation and the FBG peak shift are physically independent signals which both carry information about the temperature.

In this paper, we integrated three methods of temperature determination in a single sensor using S-FBG, thermal radiation, and a conventional type B thermocouple. Using fixed-point cells, this sensor was calibrated and the temperature measurement uncertainty determined. We report on the results of embedding such a sensor in an industrial process at a silicon foundry at Elkem ASA Technology (Norway), where it was subjected to repeated heating and cooling cycles over a period of more than 3 weeks in a silicon re-solidification furnace. The thermocouple was also calibrated and used as a well-known in situ traceable reference for the optical methods.

Section 2 presents the background for the signal generation and subsequent data analysis for the sensor. An analysis on the reliable determination of the spectral position is shown. Section 3 describes the manufacturing process of the hybrid sensor, with an overview of the entire readout system that was used. The characterization and calibration of the sensor elements is explained in Section 4, including measurements in an induction furnace to assess the effect of scattered and absorbed light. Using the calibrated hybrid sensor, an industrial production process of silicon in a high-temperature furnace was monitored in Section 5. The results of all three sensor principles (thermocouple, FBG, thermal radiation) are compared, and the stability of the sensors is determined. We summarize and discuss our findings in Section 6, where we also offer our views on the outlook of implementing a data merging process to enhance robustness and precision of the temperature recording.

## 2. Theory

### 2.1. The Sapphire Fiber Bragg Grating

The relevant optical and geometric properties of the sapphire fiber we use are summarized in Table 1. The normalized frequency *V* is nearly 300 for wavelengths around 1.55 µm, which is more than two orders of magnitude larger than the limit for single-mode transmission [22,23]. The transmission of more than 30,000 modes has important consequences for the interpretation of the reflected Bragg signal. The distinct peak, known from single-mode FBGs, becomes a rather wide and asymmetric shape [17]. This can be understood by considering the Bragg condition for the wavelength $\lambda_{B}$ for a periodic perturbation in the fiber [24]:

(1) $$\lambda_{B}=2n_{\mathrm{eff}}\Lambda .$$

Λ represents the grating period and $n_{\mathrm{eff}}$ is the effective refractive index of the guided modes of the waveguide. Due to the extreme high number of guided modes, the mode indices can be assumed as equally distributed from 1.74 the sapphire refractive index down to a refractive index of 1.0 of fiber surrounding air, whereas higher modes have a smaller refractive index. Hence, the Bragg wavelength shifts slightly for each optical mode, causing an extreme broad spectrum—theoretically several hundred nanometers. However, on the other side, higher modes have more field intensity close to the fiber’s surface and are, therefore, more strongly attenuated. This dramatically reduces the number of guided modes. Both effects, the large number of guided modes and the increasing attenuation for higher modes, result in an asymmetric peak (see Section 2.2) with a steep long-wavelength edge (fundamental mode) and flat, falling short-wavelength flank (increasing attenuation for higher modes).

**Table 1:** Relevant properties of the used 100 µm sapphire fiber.

Refractive index $n_{\mathrm{core}}$ [22,25]|$n_{\mathrm{clad}}$	1.74 | 1
Numerical aperture NA *	1.42
Normalized frequency *V* ($\lambda_{\min}$ = 1550 nm) *	289
Mode volume *M* *	> 30,000
Max. angle of propagation $\theta_{\max}$ *	55.2°
Thermo-optical coefficient $\alpha_{n}$ [22]	$12\times 10^{-6}$ K^−1^
Thermal expansion coefficient $\alpha_{\mathrm{th}}$ [22]	$7.15\times 10^{-6}$ K^−1^
Melting point [22]	2072 °C

* calculated according to [22,23].

Since the fiber expands with temperature, both the grating period and the refractive index change with temperature. The change of the center, or Bragg wavelength $\Delta \lambda_{B}$, can be expressed as a linear function of temperature and strain [24]:

(2) $$\frac{\Delta \lambda_{B}}{\lambda_{B}}=\left(1-p\right)\varepsilon +\left(\alpha_{th}+\alpha_{n}\right)\Delta T,$$

where ε is the applied strain, *p* the effective Pockel-coefficient, $\alpha_{th}$ the thermal expansion coefficient, and $\alpha_{n}$ the thermo-optical coefficient. Due to the strain decoupling in the sensor design, we assume in the following that strain effects are negligible for the temperature determination.

### 2.2. Analysis of the S-FBG Signal

**Figure 1:**
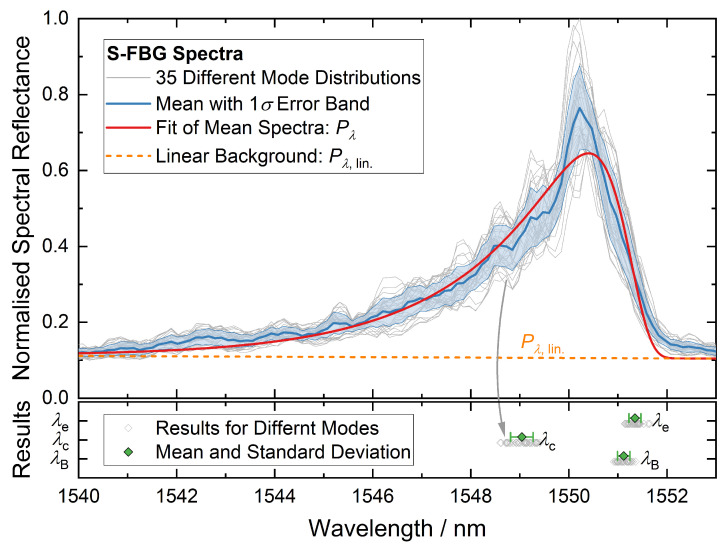
Characteristic asymmetric peak of the S-FBG and the results of different methods of spectral position determination. Gray lines show a set of 35 single measurements. The blue line represents the mean spectra with 1σ error band. The red line is the fit function of the mean spectra $P_{\lambda }\left(\lambda \right)$. Gray symbols show the three wavelength features $\lambda_{B}$, $\lambda_{c}$, and $\lambda_{e}$ for each measurement, while the green indicate the mean position with standard deviation (for details, see text).

Surface imperfections of the fiber further exacerbate the issue of multimodal transmission because there may be a mode-dependent energy loss. The effect is to add spikes in the spectrum, akin to noise but temporally stable if the fiber and readout system is fixed in place. Figure 1 shows examples of spectra received. This makes the precise and reproducible determination of the spectral position of the S-FBG peak challenging. In the literature, curve fitting was often used for this purpose [11,14,26]. We extend this curve fitting method using a linear background and an analytical expression for the long-wavelength edge of the peak.

In the following, three different methods for determining the position were compared (defined in the boxed equations): Bragg wavelength of a peak fit ($\lambda_{B}$, Equation (3)), centroid detection ($\lambda_{c}$, Equation (4)), and long-wavelength edge of the peak ($\lambda_{e}$, Equation (8)). For the evaluation, a Gaussian-like fit function was used, which approximates the spectral power $P_{\lambda }$ of the sapphire FBGs well [26] (see also Figure 1):

(3a) $$\begin{array}{cc} P_{\lambda }\left(\lambda \right) & =P_{\lambda ,\;\mathrm{peak}}\left(\lambda \right)+P_{\lambda ,\;\mathrm{lin}.}\left(\lambda \right),\;\mathrm{with} \end{array}$$

(3b) $$\begin{array}{cc} P_{\lambda ,\;\mathrm{peak}}\left(\lambda \right) & =a\;\cdot \;\exp\left[\frac{\lambda -\lambda_{B}}{w_{1}}-\exp\left[\frac{\lambda -\lambda_{B}}{w_{2}}\right]\right], \end{array}$$

(3c) $$\begin{array}{cc} P_{\lambda ,\;\mathrm{lin}.}\left(\lambda \right) & =P_{0}+m\;\cdot \;\left(\lambda -\lambda_{B}\right). \end{array}$$

where $P_{0}$ and *m* are the parameters of the linear background $P_{\lambda ,\;\mathrm{lin}.}$. The peak shape $P_{\lambda ,\;\mathrm{peak}}$ is defined by its height parameter *a*, and its width or slope of the edges characterized by $w_{1}$ and $w_{2}$. The Bragg wavelength $\lambda_{B}$ can be used as a parameter to track the temperature-dependent wavelength shift [11,14,26]. The fit is also used to extract a background-compensated centroid $\lambda_{c}$. With *y* the measured data minus the linear background $P_{\lambda ,\;\mathrm{lin}.}$, $\lambda_{c}$ is calculated numerically from the spectrum:

(4) $$\lambda_{c}=\frac{1}{\int y(\lambda )d\lambda }\int y(\lambda )\lambda d\lambda .$$

However, as will be shown in Section 5.2, the width of the reflection peak can change with aging of the sensor, while the edge toward longer wavelengths remains almost unchanged. This motivates the construction of a third estimator for a distinctive wavelength, the edge wavelength $\lambda_{e}$ defined as the intersection point of the peak from Equation (3b) with 1/e of its height (see also Figure 1).

We deduce an analytical expression for this edge wavelength as follows. With the simplification $x=\lambda -\lambda_{B}$ and $z=P_{\lambda ,\;\mathrm{peak}}\left(\lambda \right)/a$, the function describing the relative peak shape is:

(5) $$z\left(x\right)=\exp\left[\frac{x}{w_{1}}-\exp\left[\frac{x}{w_{2}}\right]\right].$$

It can be shown that the maximum point $\left(x_{\max},z_{\max}\right)$ is:

(6) $$\left(x_{\max},\;z_{\max}\right)=\left(w_{2}\ln\left[\frac{w_{2}}{w_{1}}\right],\;\;{\left(\frac{w_{2}}{w_{1}}\right)}^{w_{2}/w_{1}}\exp\left[-\frac{w_{2}}{w_{1}}\right]\right).$$

The intersection point $x_{h}$ with an arbitrary high *h* is defined by $z\left(x_{h}\right)\overset{!}{=}h$ and leads to:

(7) $$x_{h}=w_{1}\ln\left[h\right]-w_{2}W_{-1}\left[-\frac{w_{1}h^{w_{1}/w_{2}}}{w_{2}}\right],$$

where $W_{-1}\left(z\right)$ is the −1 branch of the Lambert W-function [27], which can be efficiently calculated (e.g., in python: scipy.special.lambertw(z, k = −1)). Mathematical solutions are W_0_ and W_−1_, i.e., the 0 and −1 branch of the Lambert W-function, where W_0_ describes the intersection with the short-wavelength flank and W_−1_ with the long-wavelength edge so that only the latter is required here. So the searched edge wavelength $\lambda_{e}$ results from $\lambda_{e}=\lambda_{B}+x_{h}\left(h=z_{\max}/e\right)$ and can be simplified with $W_{-1}\left[e\right]=1$ and $W_{-1}\left[-1/e\right]=-1$ to:

(8) $$\lambda_{e}=\lambda_{B}+w_{2}\left(\ln\left[\frac{w_{2}}{w_{1}}\right]-1-W_{-1}\left[-\exp\left[-\frac{w_{1}}{w_{2}}-1\right]\right]\right)-w_{1}.$$

Figure 1 shows an example of extracting the three wavelength features $\lambda_{B}$, $\lambda_{c}$, and $\lambda_{e}$ from spectra recorded in a Cu fixed point cell. A total of 35 spectra were recorded using a different setting of the mechanical mode mixer ((3c) in Section 3.3), which slightly changed the multimode spectrum. Preliminary tests had shown that this type of mechanical manipulation of the spectra is representative of the fluctuations of the S-FBG spectra due to external influences, like sensor transport (for details, see Section 5.2). The blue curve shows the mean spectrum of all 35 individual measurements with the standard deviation as error band.

As can be seen from the variation in the spectral positions in Figure 1, the centroid wavelength varies around twice as much as the fitting methods (see Table 2). $\lambda_{e}$ shows the smallest variation with 0.122 nm or approx. 4 K (see Table 2). As discussed in Section 5.2, $\lambda_{e}$ also provides the highest stability even after the field trial (see Section 5.2). Hence, we have used this wavelength in the analysis of S-FBG data in the rest of the paper.

### 2.3. Planck Radiation Sensing

Thermal radiation is described from first principles by the Planck distribution of ideal blackbody radiation. However, its detection and subsequent interpretation as a temperature is affected by a number of physical influences, from imperfect optical detection systems to non-ideal properties of the object under consideration. In particular, spectral transmission and stray light in the optical detection system affect the temperature dependence of the detected signal. However, the Sakuma-Hattori equation is a commonly used approximate function to relate temperature and received signal [28]:

(9) $$S\left(T\right)=\frac{a_{1}}{\exp\left[c_{2}/\left(a_{2}T+a_{3}\right)\right]-1},$$

where $c_{2}=hc/k_{B}$ is the second radiation constant, and the constants $a_{i}$ are determined by a curve fit to calibration points. The Sakuma-Hattori equation tracks the signal vs. temperature behavior with high fidelity, and the fitting procedure automatically considers certain aspects of the optical detection system. While the Sakuma-Hattori equation, in principle, provides a precise and traceable temperature measurement considering the device has been calibrated, e.g., at an national metrology institute [29], in practice, there are two major issues which compromise the calibration, which are investigated practically in Section 4.1.

Firstly, in the present work, we used the fiber without a coated tip. The purpose of the coating is to establish a consistent blackbody-like object that is identical in the lab and in the deployed scenario. However, the high temperatures involved, as well as the fragility of the FBG inscription process, rendered it impractical to apply a coating to the tip in our case. See [20] for more detailed information on cladding strategies for sapphire fiber and technical challenges. Hence, the thermal radiation we measured originates from different objects in the calibration laboratory and in the field test.

Secondly, some light energy is lost through absorption and surface imperfections. This is particularly prevalent with sapphire due to the non-circular fiber cross section. Furthermore, the fiber itself emits thermal radiation, so the temperature profile along the fiber also influences the signal emerging at the fiber end. Spectrally resolved detection could, in principle, be used to reconstruct a temperature profile along the fiber using advanced inversion algorithms [30]. In our specific case, both the limited bandwidth available and the fact that stray light is admitted by the fiber conspire against the effectiveness of a reconstruction procedure. The stray light could have been alleviated with a proper coating, but, again, the temperatures involved make it difficult to identify a suitable coating material (see [20]).

However, while the absolute temperature reading is challenging to transfer from the calibration laboratory to the field, once the sapphire fiber is installed in its intended place, the thermal radiation data represents a consistent, stable, and reliable sensor, as will be shown later. For simplicity, we will use the term Planck radiation to describe the thermal radiation detected at the spectrometer, even though it does not strictly follow the Planck distribution from an ideal blackbody.

## 3. Fabrication of the Hybrid Sensor and Experimental Setup

### 3.1. S-FBG Inscription

A detailed explanation of the inscription process can be found in Elsmann et al. [14,22]. Since sapphire itself is not photosensitive under illumination with moderate intensities, a Ti:Sa-fs-laser system was used for the purpose. First-order gratings require a period length of roughly 450 nm, and, hence, the frequency-doubled light of a regenerated-amplified Ti:Sa-fs-laser system was used. Specifications of the laser are: repetition rate 1 kHz, wavelength 400 nm, 540 mW averaged power, beam-diameter of 8 mm.

To form the grating pattern, a two-beam phase-mask interferometer was used. Such a symmetric interferometer uses a phase mask (grating with 888 nm pitch) to split every pulse in two. Each of the beam arms is reflected at mirrors and then interfere at the position of the fiber. The setup enables fine-tuning of the interference period and, therefore, the Bragg wavelength by rotating the mirrors. To achieve sufficient photon density inside the sapphire fiber, the pulses are focused into a line with a cylindrical lens. The focal line width is in the order of 15 µm, which is scanned across the fiber at low speed (scanning speed 0.1 µm/s).

### 3.2. Design of the Hybrid Sensor Element

**Figure 2:**
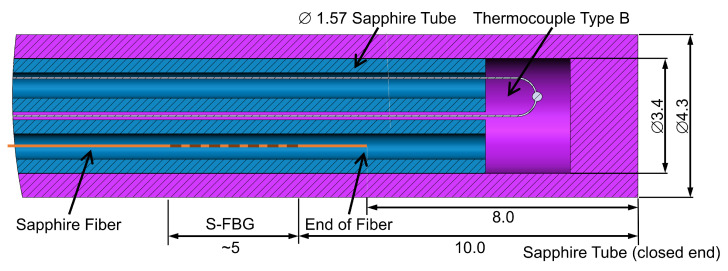
Sketch (dimensions in mm) of the sensor tip with the placement of its inner elements, including type B thermocouple and sapphire fiber.

The tip of the sensor package is shown in detail in Figure 2. The outer shell is a sapphire tube with a sealed cap. Inside the protective tube, two other thin sapphire tubes are inserted. One of them contains the optical fiber, and the other is used as electrical insulation for one of the thermocouple wires. The other wire is placed directly in the protective tube, and the two wires are welded together close to the sealed cap. The installed thermocouple is a type B (70% Pt/30% Rh–94% Pt/6% Rh), which can be used up to 1700 °C [31].

The distance between the thermocouple weld and the S-FBG is around 10 mm. Due to the fact that the fiber is uncoated, the collection of thermal radiation differs from that of a well-defined blackbody, as described in Section 2.3. This causes an high uncertainty in the traceability. However, as shown in Section 5, referencing directly under process conditions is possible and allows reliable and low-noise temperature measurements.

### 3.3. Experimental Setup

**Figure 3:**
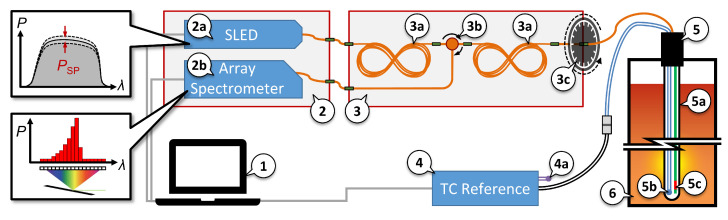
Schematic of the setup during the field measurements at Elkem ASA Technology and the calibration at PTB. The orange connections are optical multimode fibers (OM3). The most important components are: (**1**) software for data acquisition and device control; (**2**) Unit with active optical components: (**2a**) superluminescent diode (SLED) with power control, (**2b**) spectrometer; (**3**) Unit with passive optical components: (**3a**) two mode scramblers, (**3b**) circulator, (**3c**) torsion-based S-FBG mode mixer; (**4**) specialized thermocouple measuring instrument with (**4a**) cold junction temperature compensation; (**5**) hybrid sensor: (**5a**) outer sapphire protection tube, (**5b**) type B thermocouple, (**5c**) sapphire fiber with FBG; (**6**) furnace.

Figure 3 shows the schematic layout of the experimental setup used for the sensor calibration at PTB and the later field test at Elkem ASA Technology. The main components of the device are the device control unit with data acquisition (1), the passive and active optical components (2, 3), the measuring instrument for thermocouples (4), and the hybrid temperature sensor (5).

The control software was used to automatically control the power of the superluminescent diode (SLED) (2a) and the integration time of the spectrometer (2b). Specifications of the spectrometer: thermoelectric cooled InGaAs photodiode array with 512 pixel, 25 µm entrance slit, 1 µm grating pich, full width at half maximum of FWHM≈0.18 nm (optical resolution), spectral range from 1529 nm to 1590 nm, 16-bit analog-to-digital converter, integration time from 1 ms to 120 s. For the interrogation of the S-FBG, the SLED operates at 98% of the maximum power, which results in around 3.5 mW at the mechanical mode mixer (3c). For the Planck measurements, the SLED was switched off by the software. The integration time for both measurement modes was software controlled so that the maximum signal was between 70% and 95% of the dynamic range of the spectrometer. The S-FBG integration time was typically between 30 ms and 40 ms, which corresponds to a sampling rate of more than 20 Hz. The integration time of the Planck radiation measurements was substantially longer at between 1 s (T≈1600 °C) and 30 s (T<850 °C).

From the factory, the output of the SLED is realized by a single-mode fiber. To excite all modes of the multimode fiber (see Table 1), a mode scrambler (3a) was used in front and after the circulator (3b). The operating principle of the mode mixer (3a) is based on a tight (14 mm radius) eight-shaped winding of the OM3 multimode fiber, in our case using around 10 m. In addition to this, a mode mixer (3c) was used at the output of (3) to the sensor. This torsion-based mechanical mode mixer consists of an approx. 10 cm long fiber section with one fixed and one 360°-rotatable end. The mode mixing results from the fiber twist and related torsion, which influences the mode guidance of the fiber. This makes it possible to manipulate the optical modes before they are fed to the S-FBG-based temperature sensor (5) and results in a manipulation of the peak shape of its reflection. Furthermore, it offers a possibility to examine the spectra specifically at different mode excitation (see Section 2.1).

The hybrid sensor (as described above) consists of an outer (5a) and two inner sapphire protection tubes. Inside these tubes are the type B thermocouple (5b) and the sapphire fiber with FBG (5c). While the reflection spectrum of the sapphire fiber is interrogated by the optical part of the setup, a specialized thermocouple measuring instrument (4) with cold junction temperature compensation (4a) was used to measure the thermocouple. Specifications of device: 0.01 K resolution; 0.001 K cold junction resolution; 1.25 Hz sampling rate; 24-bit analog-to-digital converter; temperature range for type B thermocouple from 250 °C to 1820 °C.

## 4. Characterization and Calibration of the Hybrid Sensor

### 4.1. Impact of a Heat Source Location along the Fiber

In order to evaluate the influence of the position of the heat source along the fiber on the optical data, we performed a series of immersion experiments on an inductively heated furnace of a fiber drawing tower. Due to the inductive heating, it was not possible to use a thermocouple for this experiment.

Two S-FBGs with a lateral dimension of approx. 0.5 cm were inscribed into the 1-m long sapphire fiber, which is embedded in a protective tube made of corundum. The distance between the sensor tip and the fiber tip, first S-FBG and second S-FBG are 1.3 cm, 3 cm, and 6 cm (see Table 3).

**Figure 4:**
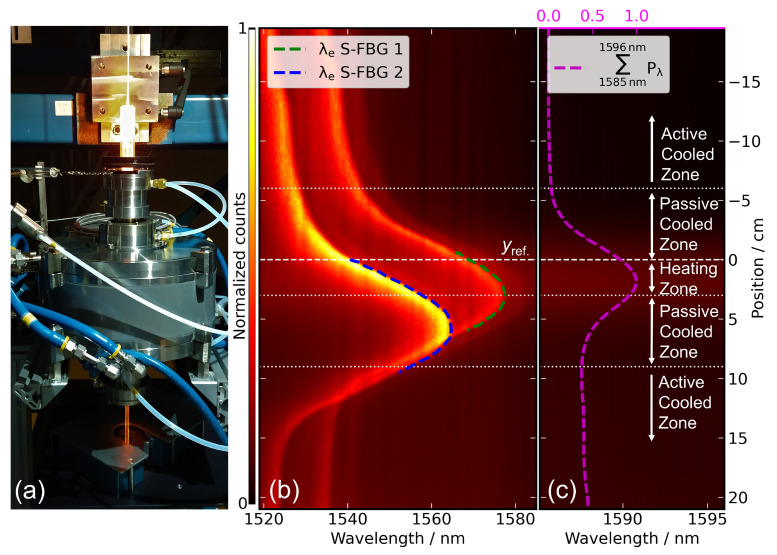
(**a**) Inductively heated furnace of a fiber draw tower. (**b**,**c**) Determined optical spectra relative to the position of the sensor in the draw tower with normalized spectrometer counts as false color visualization. (**b**) Spectrum between 1519 nm and 1585 nm with the two S-FBGs. The *green* (S-FBG 1) and *blue* (S-FBG 2) line are the determined edge wavelength $\lambda_{e}$ according to Equation (8). (**c**) Spectrum from 1585 nm and 1596 nm used for the thermal radiation-based data processing. The *magenta* line represents the normalized summed spectral power.

Figure 4a shows the furnace of the draw tower, which basically consists of a 3 cm long heating zone under argon atmosphere (in Figure 4b,c approx. from position 0 cm to 3 cm) with an adjacent 6 cm passive cooled zone followed by a 12 cm active water cooled zone. The relatively small heating zone and the slightly inward-oriented gas flow ensure a high-temperature gradient between hot and cold zones. In the start position of the experiment, the protection tube tip was placed directly above the upper cooling zone (−19.5 cm). The end position (approx. 20.9 cm) was chosen so that both S-FBGs nearly reached the end of the lower cooling zone. The fiber was lowered and lifted with a travel speed of 0.2 cm/min for the experiment. Figure 4b,c shows the results of lowering the sensor as spectral intensity (false color plot) as a function of fiber position and wavelength. The position of the S-FBGs peaks (see Figure 4b, blue and green lines) were determined by $\lambda_{e}$ (see Equation (8)). For the thermal radiation, the summed intensity (further named Planck) from 1585 nm to 1596 nm (see Figure 4c, magenta line) was used. The plateau’s center of these lines were determined relative to the reference position $y_{\mathrm{ref}.}$ (at 0 cm), where the sensor tip enters the heating zone of the furnace. The following Table 3 summarizes the results.

As can be seen, the thermal radiation reaches its maximum first at approximately 1.8 cm or 0.5 cm after the end of fiber passed the furnace center. This maximum of collected thermal radiation results from a superposition of several individual components: coupling and loss of thermal radiation through the side walls and the tip of the fiber, along with the emission from the hot fiber itself. This position is reached shortly before the protection tube tip leaves the heating zone, while the fiber tip already passed the center of the furnace. The thermal radiation collected by the fiber tip is forming the peak in Figure 4c as it contributes dominance compared to sidewall and self-radiation. Since the transmission of the radiation takes place both in the direction toward the detector and the end of the fiber, a part of the intensity is coupled in and out at the end of the fiber. After the fiber tip leaves the heating zone, only the sidewall coupling and the self-radiation contribute to the detected intensity. The remaining intensity (see Figure 4c, magenta line) represents the self-radiation and sidewall coupling contributions, which cause the linear increasing background from 9 cm onward in Figure 4c. This results from the decreasing transmission losses (approx. 5 dB/m [22]), as the distance between heater and detector decrease. The fundamental basic problem that the measured value depends on the temperature distribution along the sensor also exists with thermocouples, although to a substantially smaller extent (see Section 4.2).

This illustrates that several factors are essential for this method of temperature determination: the length and position of the heating zone together with its temperature profile. A suitable coating material or cap in the relevant section of the fiber would help to reduce the effects of temperature gradients along the fiber. Although variations due to scattered light and absorption effects would still be present, we assume that calibration would be possible within a useful uncertainty budget.

In contrast, the S-FBGs responds to the actual temperature at their location in the fiber. As expected, the distance between both plateau center positions is approx. 3 cm, corresponding to the spatial distance between the S-FBGs. A comparison of the corresponding longest wavelengths, indicating the highest temperature, shows a difference of 12 pm to 60 pm between lowering and lifting (see Table 4). This corresponds to a difference of less than 2 K, taking a sensitivity of 30 pm/K into account, which is in the range of the expected temperature fluctuations of the draw tower furnace.

### 4.2. Calibration of the Reference Thermocouple 

As described in Section 3.2, the hybrid sensor includes a type B thermocouple. This was necessary for the process control of the industrial furnace and further serves as a reference for the optical methods. The thermocouple was calibrated in parallel with S-FBG spectra at fixed points traceable to the International Temperature Scale (ITS-90) [32], according to international guidelines [33]. These fixed points are the freezing points of high-purity metals, which provide a very homogeneous, stable, and precisely known temperature. In our case, the thermoelectric voltage was measured simultaneously with the direct reading thermocouple measuring instrument of the portable setup (see Section 3.3) and a calibrated high-precision digital multimeter. In this way, we could also check the temperature reading with cold junction compensation ((4a) in Section 3.3) in comparison to a cold junction in the ice point. The six used fixed points reaching from roughly 420 °C to 1490 °C (Table 5). During the field trial, the temperature was measured with a specialized thermocouple measuring instrument, which calculates the temperature $T_{\mathrm{TC}}$ according to the type B reference function [34]. The calibration results in a constant offset to the ITS-90 $T_{90}$:

(10a) $$\begin{array}{cc} T_{90} & =T_{\mathrm{TC}}+\left(1.2\pm U_{k=2}\left(T_{\mathrm{TC}}\right)\right)\;K,\;\mathrm{with} \end{array}$$

(10b) $$\begin{array}{cc} U_{k=2}\left(T_{\mathrm{TC}}\right) & \approx \left\{\begin{array}{cc} 1.5\;K & \mathrm{for}\;T=250\;{}^{\circ }C\ldots 1100\;{}^{\circ }C\\ 0.005\;K/{}^{\circ }C\;\cdot \;T_{\mathrm{TC}}-4\;K & \mathrm{for}\;T=1100\;{}^{\circ }C\ldots 1600\;{}^{\circ }C. \end{array}\right. \end{array}$$

So the temperature $T_{90}$ is believed to lie in the interval defined by expanded uncertainty $U_{k=2}\left(T_{\mathrm{TC}}\right)$ with a level of confidence of approximately 95%.

An overview of the uncertainty contributions is shown in Table 5, which is dominated by the inhomogeneity of the thermocouple. In a thermocouple, the thermoelectric voltage is generated along the length of the wires wherever temperature gradients exist. Thermoelectric inhomogeneities are position-dependent variations in the Seebeck coefficient [35]. The most common causes of inhomogeneity are cold work and strain, vacancy effects, ordering effects, oxidation, and contamination, with most thermocouple types suffering from all effects to some degree. Thus, the measured temperature depends slightly on the temperature profile along the sensor and not only on the temperature at its tip. Inhomogeneities are measured by progressively passing the thermocouple through a sharp temperature gradient and monitoring changes in the voltage produced by the thermocouple [36,37]. The uncertainty due to inhomogeneity along the electrical wires is mostly the dominant source of uncertainty in thermocouple measurements, which is also the case here (see Table 5).

The calibrated thermocouple is also used to calibrate the S-FBG spectra at temperatures in addition to the fixed points (see Section 4.3) and for the in situ calibration of the Planck measurements during the field trial (see Section 5.1). The calibration of the S-FBG at temperatures above the fixed points used here is possible due to the known reference function [34], to which the calibration is only a small correction (see Equation (Section 4.2)).

### 4.3. Calibration of the S-FBG Sensor

**Figure 5:**
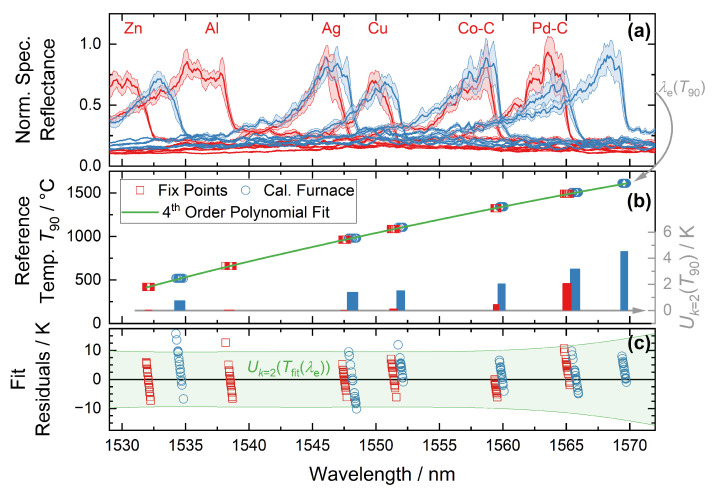
(**a**) Mean spectra of the sapphire fiber for different calibration temperatures with 1σ error band. *Red* curve corresponds to a measurement performed in a vertical ITS-90 fixed point and *Blue* to the horizontal calibration furnace. (**b**) Results of the analytical edge detection ($\lambda_{e}$) for all recorded spectra. The corresponding temperature arises from the fixed point and the calibrated thermocouple of the sensor (see Section 4.2), respectively. The *green* curve is the 4th order polynomial fit of those measurements, weighted by the temperature uncertainty. The *right axis* shows the expanded uncertainty of the calibration temperatures as a bar chart. (**c**) Fit residuals of each calibration point and resulting expanded combined uncertainty of the temperature resulting from the S-FBG spectra $U_{k=2}\left(T_{\mathrm{fit}}\left(\lambda_{e}\right)\right)$.

As introduced in Section 2.2, three different methods ($\lambda_{B}$, $\lambda_{c}$, $\lambda_{e}$) for determining the position of the S-FBG peak were investigated. However, only the latter is used here for calibration and field measurements, as it is the most stable and precise (see Section 5.2). The results of the calibration are summarized in Figure 5. The S-FBG spectra were recorded both in the fixed points of the ITS-90 (red) and in a horizontal calibration furnace, with the previously calibrated thermocouple as reference (blue). In addition to the calibration point at roughly 1600 °C, the measurements in the calibration furnace are used to investigate the influence of the installation orientation of the sensor. As Figure 5c shows, there is no significant difference between the measurements in the vertical fixed points and the horizontal calibration furnace. Thus, both the assumption of the stress-free fiber and the horizontal use in the field test are ensured.

Figure 5a shows the mean spectra of the sapphire fiber for different calibration temperatures with 1σ error band. This mean value and the standard deviation result from 35 individual measurements. The angle φ of the torsion-based mode mixer (see Figure 3c) was varied in each case, $\varphi =[0^{\circ },20^{\circ },\ldots ,340^{\circ },320^{\circ },\ldots ,0^{\circ }]$, to examine the spectra specifically at different mode excitations. The random nature of the mode transmission and the accuracy of the angle setting lead to non-identical modes at the same angle on the up and down paths.

Results of the analytical edge detection ($\lambda_{e}$) for all recorded spectra are shown in Figure 5b with their corresponding temperatures. These temperatures arise from the fixed point value [32] and the comparison with the calibrated thermocouple of the sensor itself (see Section 4.2). The right axis shows the expanded uncertainty of these temperature values.

As expected from Section 4.2, they are significantly larger for the measurements in the horizontal calibration furnace referencing to the thermocouple. The calibration function $T_{\mathrm{fit}}\left(\lambda_{e}\right)$ is calculated as a 4th order polynomial, where the fit was weighted by the temperature uncertainties. The resulting sensitivity is between 26 pm/K at 500 °C and 39 pm/K at 1600 °C, which is comparable to other published data [11,14,17,18,26].

Figure 5c shows the resulting residuals of each calibration point. The green area is the expanded total uncertainty of the calibration function $U_{k=2}\left(T_{\mathrm{fit}}\left(\lambda_{e}\right)\right)$, of which the contributions are shown in Table 6. The total uncertainty results mainly from the standard deviation of the fit residuals, which arise from variations in the transmitted S-FBG modes (see Section 2.2). At temperatures above 1400 °C, the rising uncertainty of the temperature values leads to a greater total uncertainty. Due to the high-temperature homogeneity of the fixed point cells and the horizontal calibration furnace compared to the small distance between thermocouple and S-FBG (around 1 cm, see Figure 2), the temperature difference between them is negligible. Variations in the wavelength scale of the spectrometer are included in the fit residuals. As shown in Section 5.2, the wavelength calibration of the spectrometer also did not change during transport to the field test.

## 5. Results of the Field Trial in an Industrial Silicon Production Facility

### 5.1. Procedure and Context of the Field Trial

Subsequent to the calibration, the hybrid sensor was tested in a field trial at Elkem ASA Technology in Kristiansand (Norway). We monitored 25 cycles of the silicon resolidication. This step of the process takes around a day, which means a total sensor operating time of over 3 weeks. During this time, the hybrid sensor remained in the production furnace, which corresponds to continuous operation of about 500 h. Within each cycle, the furnace reaches temperatures up to 1600 °C to ensure an appropriate silicon purity. The furnace is first heated up to above the melting point of silicon (1410 °C). After a stabilization period, the furnace is filled with the approx. 1600 °C hot silicon melt. Afterward, a slow, controlled cooling of the melt to room temperature is carried out. Precise process control and, thus, monitoring of the temperature is essential to reduce material impurities of the silicon. Normally, this process is monitored by several thermocouples. Since our calibrated hybrid sensor is compatible with industrial process monitoring, it was possible to replace one of the regular sensors with our prototype. Comparable to the regular monitoring sensors, the hybrid thermometer is installed in a protective sleeve and is, thus, not directly exposed to the silicon in the furnace chamber. This protective sleeve is located near one of the heating elements of the furnace. The distance from the hot end to the cold end of the sensor is slightly less than 1 m. Since the furnace wall is actively cooled, the cold end of the thermometer is approximately at room temperature. As a result, the temperature gradients along the sensor can be quite high, especially when the heating elements are operated at full power.

Within the field trial, we treated the thermocouple as an in situ, known reference. After these 25 process cycles, the sensor was demounted and transported to PTB Berlin (Germany) for recalibration at fixed points to investigate its stability.

**Figure 6:**
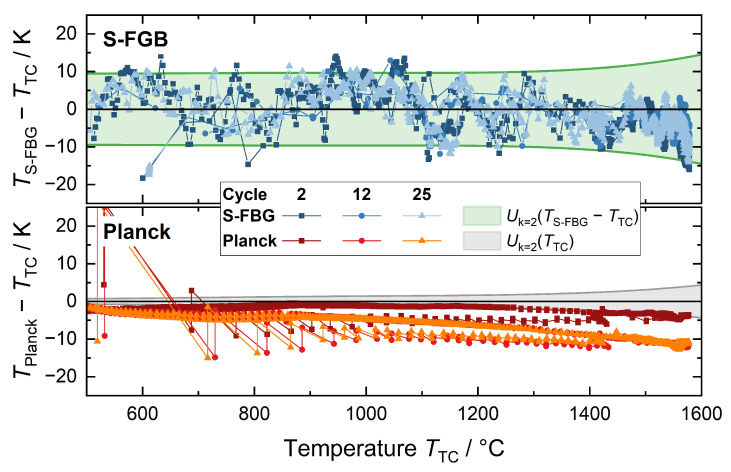
Deviations between the different temperature readings of the hybrid sensor for three representative cycles. *S-FBG/blue*: Difference between the calibrated thermocouple and the calibrated S-FBG together with their expanded combined uncertainty (*green area*). *Planck/red*: Difference between the thermal radiation (Planck) and thermocouple measurements. For the Planck data, the Sakuma-Hattori equation was fitted to the data points of the first cycle, using the thermocouple readings as the reference temperature. The *grey area* represents the expanded uncertainty of the thermocouple.

Figure 6 shows the temperature differences (for cycle 2, 12, and 25) between the optical methods (S-FBG and Planck) and the determined temperature by the thermocouple. The green area represents the expanded combined uncertainty of the S-FBG sensor and the thermocouple determined during their calibration, while the grey area represents the uncertainty of the thermocouple alone, for comparison. Each of the data points shown represents one measurement during a production cycle. Due to the holding times in the process at temperatures above 1400 °C, the number of data points for this temperature range is higher.

As explained in Section 4.1, the calibration of the Planck emission was not possible due to the dependency of detected thermal radiation to the spatial thermal profile of the furnace. Therefore, the first cycle was used to fit the Planck data to the Sakuma-Hattori equation as described by Equation (9). For this purpose, 20 data points covering the entire temperature range (500 °C to 1550 °C) are picked arbitrarily to adjust *S* to $T_{\mathrm{TC}}$. The resulting fitted constants are $a_{1}=3.4561\times 10^{5}$, $a_{2}=1.5351$, and $a_{3}=2.6813$, which were determined with a fitting uncertainty (k=2) below 4 K for temperatures above 800 °C. It should be noted that the exact values of the constants may change slightly depending on the selection of points. However, this has little effect on the fitting uncertainty, as this is determined by the uncertainty in the thermocouple calibration together with the scattering of points used for the fitting.

As can be seen in the upper part of the Figure 6, most of the measured values are within the expanded combined uncertainty. A detailed analysis of all recorded data points (>100,000) shows that 96% of them are within this uncertainty. This fits well to the estimated 95% coverage of the expanded combined uncertainty and is a good indication for the correct estimation of the respective uncertainties.

Comparable to the calibration (see Figure 5b), the determined temperature differences between S-FBG and thermocouple show a trend for temperatures above 1400 °C toward lower determined temperatures by the S-FBG. We think this is a combined effect of higher uncertainty of the thermocouple with increasing temperature and a sensitivity change of the S-FBG with temperature. Although this small trend is within the measurement uncertainty, it might be possible to reduce the effect in the future using additional calibration points (at higher temperatures) with ideally lower uncertainty.

The scatter of the S-FBG temperature can be explained by changes of the peak shape comparable to Figure 1. Considering the 2σ deviation (about 8 K) of our peak fit test for different mode propagation conditions (see Table 2), the fluctuations are within the expected range. In this case, the change in peak shape has a different cause. The first reason is that the multimode profile of the S-FBG moves from detector pixel to pixel, which results in amplitude changes. Second, the propagation conditions (transmission losses) of the individual modes within the gradient index fiber change slightly with temperature.

In the comparison to the temperature determination with the S-FBG, the evaluation of the thermal radiation shows a smaller point-to-point variation, which could be defined as $\left|0.5\;\left(\Delta T_{k-1}+\Delta T_{k+1}\right)-\Delta T_{k}\right|$ for the difference $\Delta T_{k}$ between the *k*-th Planck and thermocouple value. It is for 95% of the values below 1 K (see Figure 6). The remaining 5% are in parts of the industrial process with rapid temperature changes compared to the integration time, which can be explained as follows. Firstly, the integration time of the spectrometer varies between 1 s and 30 s (see Section 3.3). Hence, during fast temperature changes, the average temperature during this period differs from the thermocouple reading recorded directly afterwards. Secondly, during these faster changes in the production process, the inhomogeneity of the thermal environment of the sensor is higher. For example, since the sensor was inserted through the heating elements, the temperature along the fiber is higher than at the tip during heating, which has a noticeable effect on the Planck signal (see Section 4.1). In contrast to the S-FBG, a small hysteresis could be observed between furnace heating and cooling. This is probably mainly due to the changing temperature profiles during heating and cooling and the previously discussed dependence on the heating or cooling rate. Further, the the temperature calculated from the thermal radiation decreases in comparison to the thermocouple from cycle to cycle, as shown in Figure 6. Hence, less thermal radiation is collected over time.

### 5.2. Stability and Repeatability

**Figure 7:**
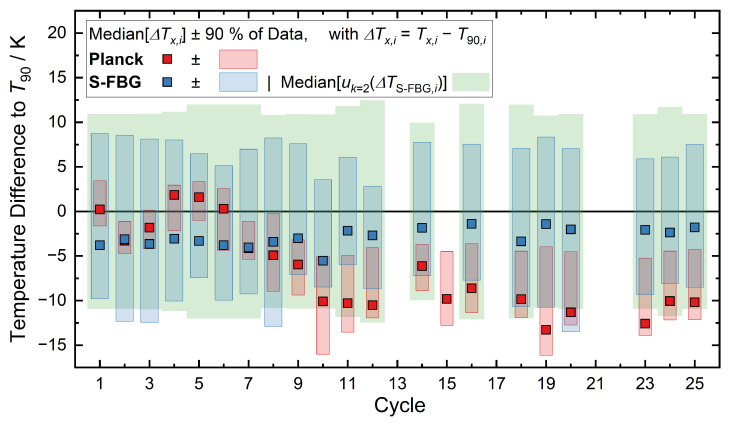
Overview of the median temperature difference between thermocouple and Planck/FBG for the 25 cycles of the field trial, which summarizes the results of over 100,000 measurements. *Blue* (S-FBG) and *red* (Planck) temperatures show the 90% range of all temperature differences determined for one cycle. The *green* bar displays the median of the expanded (k=2) combined uncertainty of S-FBG sensor and thermocouple for each cycle.

A summary of the results of all cycles of the trial are shown in Figure 7, which includes the results of over 100,000 measurements. The squares represent the median temperature difference between the optical determined temperatures (S-FBG in blue, Planck in red) compared to the thermocouple. Because of the uneven distribution of the data points over the temperature range (see Figure 6), the median provides a better representation of the typical values than the mean. As in Figure 6, the green area represents the expanded (k=2) combined uncertainty of S-FBG sensor and thermocouple. Since this uncertainty varies during a cycle, the median of the data is shown here. The blue (S-FBG) and red (Planck) temperature ranges cover 90% of the data points in each cycle. Due to an error of the SLED power supply, the data of cycles 13, 17, 21, and 22 are not properly analyzable.

The S-FBG data exhibits a consistent scatter around the simultaneous thermocouple readings of around ±8 K for 90% of the data points. It can be seen that the median deviation is always negative (−1.4 K to −5.5 K), which is consistent with the more positive residuals of the calibration fit for temperatures above 1500 °C (see Figure 5). As indicated by the smaller red bars, the Planck data scatters slightly less (between ±2 K and ±6 K). It is evident that the S-FBG sensor remains stable, while the Planck sensor drifts between cycles 5 and 10, before stabilizing. While we have not investigated the cause of this in detail, a likely explanation is the buildup of a thin, opaque layer on the outer surface of the sapphire protection tube. The layer partly blocks radiation originating at the bottom of the thermometer well, and, hence, the amount of thermal radiation transmitted into the fiber changes.

From previous experience at Elkem, the likely substance is aluminium nitride, which tends to form slowly at exposure to the temperatures involved. Unfortunately, it was not possible to evaluate this assumption within the scope of the study, since a non-destructive analysis of the material composition of the surfaces was not possible.

**Figure 8:**
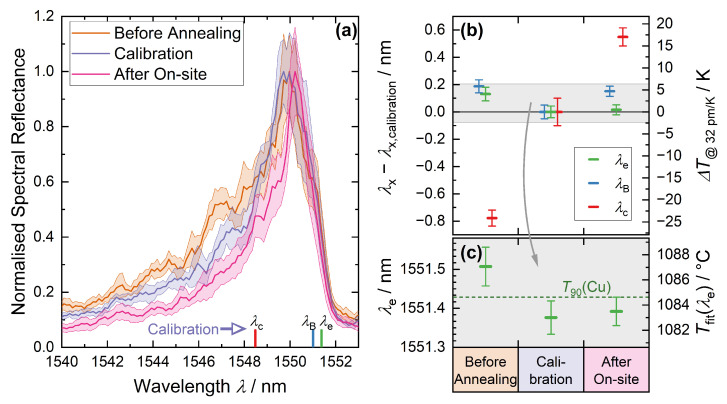
Stability and repeatability of the S-FBG spectra and its wavelength features $\lambda_{B}$ (*blue*), $\lambda_{c}$ (*red*), and $\lambda_{e}$ (*green*) (see Section 2.2) at the Cu fixed point before an initial annealing step at 1700 °C (*orange*), after this annealing and during the calibration (*violet*), and after the on-site trial (*pink*): (**a**) average spectra with standard deviation of 35 individual spectra (see Section 4.3) and position of the wavelength marker for the calibration run; (**b**) change of the mean of the 35 analyzed spectra and its standard error (error bars $=2\sigma_{\bar{x}}$); (**c**) absolute change of $\lambda_{e}$ and corresponding temperature (resulting from the calibration fit) compared to the fixed point temperature $T_{90}\left(\mathrm{Cu}\right)=1084.62$ °C.

In order to investigate the stability and repeatability of the S-FBG spectrum, the sensor was repeatedly measured at the Cu fixed point, whose absolute temperature is very well known: (1084.62±0.12) °C. Figure 8 shows the results in three different aging phases of the sensor:

1. Before high-temperature annealing of the sensor at about 1700 °C for 1 h;
2. After annealing, which is used for the calibration described in Section 4.3;
3. After the field test described in Section 5.1.

We further investigated the effects of aging on the methods used to determine the spectral position of the S-FBG (as described in Section 2.2). Figure 8a shows the mean spectra of 35 acquired measurements together with the 1σ error band. In addition, the mean position of the three determined wavelengths for $\lambda_{B}$, $\lambda_{c}$, and $\lambda_{e}$ are shown for the spectrum of the calibration. Each of the 35 spectra were obtained as described in Section 4.3 by rotating the torsion-based mode mixer. It is evident that the spectral width of the S-FBG peak has decreased due to aging. This effect appears predominant at the short-wavelength flank of the S-FBG peak.

As discussed in Section 2.1, the short-wavelength flank of the S-FBG spectrum represents modes with lower effective refractive index compared to the fundamental mode. These modes carry a higher proportion of their intensity in the outer region of the fiber, and the fact that they are suppressed indicates some form of structural change on or near the surface of the fiber.

The effect of these changes in the spectrum to the determined spectral position is shown in Figure 8b for each method. The right axis gives the corresponding temperature difference before annealing and after on-site test, relative to the calibration. To convert the wavelength changes to temperature, the sensitivity determined from the calibration of 32 pm/K around the Cu fixed point was used. Figure 8c shows the absolute change of $\lambda_{e}$ and the corresponding temperature (resulting from the calibration fit) compared to the fixed point temperature. The aging did almost not affect the long-wavelength edge detection, which demonstrates the robustness and reliability of the algorithm.

Table 7 shows the observed wavelength and temperature changes between calibration and follow-up measurement after the field trial. As shown in Figure 8, the change in the short-wavelength flank mainly affects the centroid ($\lambda_{c}$) with around 550 pm or 17 K drift caused by the on-site trial. In contrast to that, the S-FBG fit wavelength $\lambda_{B}$ (152 pm or 4.7 K) and the long-wavelength edge $\lambda_{e}$ (16 pm or 0.5 K) show higher stability. $\lambda_{e}$ shows the lowest drift and highest robustness, which supports its use for calibration and field measurement. Hence, the S-FBG signal has remained stable over the field measurements within the uncertainties of this comparison, and the same is true for the thermocouple.

This uncertainty at nearly constant temperature ($2\sigma (T_{\mathrm{Cu}})\leq 0.12$ K) is much smaller than for the calibration of the sensor, since here an averaging over 35 spectra was performed so that even uncertainties below the resolution of the spectrometer (FWHM≈180 pm) are possible. The standard error of the mean of the two measurement series and $\sigma (T_{\mathrm{Cu}})$ contribute to the total uncertainty. The total uncertainty of the thermocouple can be assessed analogously to Table 5, excluding the contribution of the residuals. The contribution of thermocouple wire inhomogeneity could not be neglected, as it may have changed during the field test.

## 6. Discussion and Conclusions

In this article, we have successfully demonstrated the metrological characterization of a high-temperature hybrid sensor using thermal radiation and calibrated S-FBG for process monitoring in harsh environments. The hybrid optical sensor additionally includes a type B thermocouple for comparison and process control.

We calibrated the S-FBG traceable to the International System of Units with an expanded measurement uncertainty of 10 K below 1400 °C and 14 K up to 1600 °C (see Section 4.3). Changes of the peak shape due to variations of the mode propagation conditions were identified to be the main reason for the measurement uncertainty of the S-FBG-based temperature measurement. Using a manually controlled mechanical mode mixer, we were able to demonstrate a possibility to minimize this behavior. Further, we derived an expression for the long-wavelength edge of the S-FBG peak $\lambda_{e}$ and show that it is much more stable against mode variations (see Section 2.2). We assume that, using the average of $\lambda_{e}$ over several spectra, the temperature uncertainty could be reduced by an order of magnitude toward the performance of the best high-temperature thermocouples. Another way to reduce the measurement uncertainty would be by reducing the number of modes (see Table 1) of the sapphire fiber as described in [12,19,20].

Furthermore, we were able to show that even long-term exposure to high temperatures (up to 1700 °C) has no negative influence on the measurement. Even after 3 weeks of repeated cycling up to 1600 °C, the S-FBG shows no significant drift. The difference between the readout before and after the field trial, analyzed at the Cu fixed point, was (0.5 ± 1.7) K and comparable to the thermocouple with (0.2 ± 2.1) K (see Section 5.2).

In contrast to the S-FBG, this multimode behavior has only a minor impact on Planck-based temperature determination. However, this temperature determination using the thermal radiation of the furnace and the fiber itself requires an in situ calibration measurement. As shown in Section 4.1, this is necessary due to the influence of the temperature profile along the sensor on its readout. When this calibration has been performed, the Planck temperature determination provides sub-Kelvin precision for slow (compared to integration time) temperature changes below, e.g., 1 K/min.

We assume that a combination of the complementary strengths of both optical sensing techniques potentially offers an even lower uncertainty. For this purpose, the temperature of the calibrated S-FBG could be used for the fitting of the thermal radiation temperature readout. The varying modes of the S-FBG can be treated as random noise for this purpose. In use, the Planck observations would be continuously recorded until the S-FBG has detected a sufficient temperature change (e.g., 200 K), after which the Sakuma-Hattori equation is fitted and also used to evaluate future temperatures. Whenever the temperature shifts far away from the original fitting range, a new fit is performed.

Based on our findings, we see a promising future for S-FBGs for high-temperature measurements. Methods are emerging that can make their accuracy competitive with thermocouples with the advantages of fiber-based sensing.

## Figures and Tables

**Table 2:** Results of the peak fitting methods for 35 different spectra at the Cu fixed point. The change of the S-FBG peak shape is induced by the mechanical torsion of a fiber with the mode mixer (3c in Section 3.3). A sensitivity of 30 pm/K has been used for the conversion of wavelength to temperature.

	Standard Deviation		
	$\lambda_{B}$	$\lambda_{c}$	$\lambda_{e}$
Wavelength/nm	0.127	0.230	0.122
Temperature/K	4.23	7.67	4.06

**Table 3:** Comparison between the geometric location of each feature and the center locations of the temperature plateaus determined by $\lambda_{e}$ for the S-FBGs and by summation of the thermal radiation in the range from 1585 nm to 1596 nm for “Planck”.

	Distance to $y_{\mathrm{ref}.}$/cm		
Determination Method	Planck	S-FBG 1	S-FBG 2
Geometrically measured	1.3 * ± 0.1	3 ± 0.25	6 ± 0.25
Center of temperature plateau during lowering ⇓	1.85	3.02	5.93
Center of temperature plateau during lifting ⇑	1.80	2.90	5.84

* fiber tip.

**Table 4:** Wavelength $\lambda_{e}$ (see Section 2.2) at the temperature plateau center for the two S-FBGs. The values were determined for lifting and lowering of the sensor through the induction furnace.

	$\lambda_{e}$ of Plateau Center/nm	
	S-FBG 1	S-FBG 2
During lowering of sensor ⇓	1577.424	1564.666
During lifting of sensor ⇑	1577.436	1564.722

**Table 5:** Overview of uncertainty contributions to the thermocouple calibration.

	Expanded Uncertainty Contributions (k=2)/K					
Fixed Point	Zn	Al	Ag	Cu	Co-C	Pd-C
Temperature/°C	419.527	660.323	961.78	1084.62	1323.85	1490.7
Fixed point temperature	0.01	0.02	0.02	0.12	0.47	2.07
Equipment & surrounding	0.22	0.14	0.10	0.10	0.08	0.08
Repeatability & stability	0.06	0.09	0.14	0.19	0.20	0.24
Inhomogeneity	0.51	0.83	1.26	1.45	1.84	2.15
Correction residuals	0.44	0.44	0.44	0.44	0.44	0.44
**Total**	**0.71**	**0.96**	**1.35**	**1.53**	**1.96**	**3.03**

**Table 6:** Overview of uncertainty contributions to the S-FBG calibration.

	Expanded Uncertainty Contributions (k=2)/K						
Fixed Point	Zn	Al	Ag	Cu	Co-C	Pd-C	- *
Temperature/°C	419.527	660.323	961.78	1084.62	1323.85	1490.7	1604.5
Temperature	0.04	0.04	0.03	0.12	0.47	2.1	4.5
Fit residuals (2σ)	9.5	9.5	9.5	9.5	9.5	9.5	9.5
λ stability,	negligible or part of the fit residuals						
*T* comparison							
**Total**	**9.5**	**9.5**	**9.5**	**9.6**	**9.9**	**11.5**	**14.0**

* comparison with calibrated thermocouple.

**Table 7:** Wavelength and temperature changes between calibration and follow-up measurement after the field trial investigated at the copper fixed point, determined for the spectral position features of the S-FBG peak and the thermocouple with their respective expanded uncertainties.

	$\lambda_{e}$	$\lambda_{B}$	$\lambda_{c}$	$T_{\mathrm{TC}}$
Δλ/pm	16±56	152±63	550±120	-
ΔT/K	0.5±1.7	4.7±2.0	17.0±3.8	0.2±2.1

## Data Availability

Not applicable.

## Abbreviations

The following abbreviations are used in this manuscript:

FBG	fiber Bragg grating
FWHM	full width at half maximum
ITS-90	International Temperature Scale (of 1990)
$\lambda_{B}$	wavelength of the S-FBG fit (defined in Equation (3))
$\lambda_{c}$	wavelength of the centroid of the S-FBG (defined in Equation (4))
$\lambda_{e}$	wavelength of the long-wavelength edge of the S-FBG fit (defined in Equation (8))
S-FBG	sapphire fiber Bragg grating
SLED	superluminescent diode
$T_{TC}$	temperature of the thermocouple

## References

[1] Hartog A.H. (2017). An Introduction to Distributed Optical Fibre Sensors.

[2] Roriz P., Carvalho L., Frazão O., Santos J.L., Simões J.A. (2014). From conventional sensors to fibre optic sensors for strain and force measurements in biomechanics applications: A review. J. Biomech..

[3] Li J., Sun X., Huang L., Stolov A. Optical fibers for distributed sensing in harsh environments. Proceedings of the Fiber Optic Sensors and Applications XV. International Society for Optics and Photonics.

[4] Chai Q., Luo Y., Ren J., Zhang J., Yang J., Yuan L., Peng G. (2019). Review on fiber-optic sensing in health monitoring of power grids. Opt. Eng..

[5] Robert G.B. (2017). Handbook of Optoelectronics: Applications of Optoelectronics.

[6] Munzke D., Duffner E., Eisermann R., Schukar M., Schoppa A., Szczepaniak M., Strohhäcker J., Mair G. (2021). Monitoring of type IV composite pressure vessels with multilayer fully integrated optical fiber based distributed strain sensing. Mater. Today Proc..

[7] (2021). IEC 61757-2-1:2021.

[8] (2020). VDE 2660-2; Technical Temperature Measurement—Optical Temperature Sensor Based on Fibre Bragg Gratings—Recommendation on Temperature Measurement and Statement of Measurement Uncertainty. 2.17 “Fibre Optic Measurement Techniques”.

[9] Lindner M., Bernard D., Heilmeier F., Jakobi M., Volk W., Koch A.W., Roths J. (2020). Transition from purely elastic to viscoelastic behavior of silica optical fibers at high temperatures characterized using regenerated Bragg gratings. Opt. Express.

[10] Rose A. (1997). Devitrification in annealed optical fiber. J. Light. Technol..

[11] Habisreuther T., Elsmann T., Pan Z., Graf A., Willsch R., Schmidt M.A. (2015). Sapphire fiber Bragg gratings for high temperature and dynamic temperature diagnostics. Appl. Therm. Eng..

[12] Ohanian III O.J., Boulanger A.J., Rountree S.D., Jones J.T., Birri A., Blue T.E. (2019). Single-mode sapphire fiber optic distributed sensing for extreme environments. Proc. SPIE 10982, Micro- and Nanotechnology Sensors, Systems, and Applications XI.

[13] Wosniok A., Skoczowsky D., Schukar M., Pötzsch S., Pötschke S., Krüger S. (2019). Fiber optic sensors for high-temperature measurements on composite tanks in fire. J. Civ. Struct. Health Monit..

[14] Elsmann T., Habisreuther T., Graf A., Rothhardt M., Bartelt H. (2013). Inscription of first-order sapphire Bragg gratings using 400 nm femtosecond laser radiation. Opt. Express.

[15] Salter P.S., Booth M.J. (2019). Adaptive optics in laser processing. Light. Sci. Appl..

[16] Fells J., Booth M., Salter P. (2020). Method of Laser Modification of an Optical Fibre. US Patent.

[17] Wang B., Niu Y., Qin X., Yin Y., Ding M. (2021). Review of high temperature measurement technology based on sapphire optical fiber. Measurement.

[18] Wilson B.A., Blue T.E. (2018). Quasi-distributed temperature sensing using type-II fiber bragg gratings in sapphire optical fiber to temperatures up to 1300 °C. IEEE Sens. J..

[19] Guo Q., Jia Z., Pan X., Liu S., Tian Z., Zheng Z., Chen C., Qin G., Yu Y. (2021). Sapphire-Derived Fiber Bragg Gratings for High Temperature Sensing. Crystals.

[20] Chen H., Buric M., Ohodnicki P.R., Nakano J., Liu B., Chorpening B.T. (2018). Review and perspective: Sapphire optical fiber cladding development for harsh environment sensing. Appl. Phys. Rev..

[21] Dils R.R. (1983). High-temperature optical fiber thermometer. J. Appl. Phys..

[22] Elsmann T. (2016). Faser-Bragg-Gitter für die Hochtemperaturanwendung. Ph.D. Dissertation.

[23] Ghatak A., Thyagarajan K. (1998). An Introduction to Fiber Optics.

[24] Kersey A.D., Davis M.A., Patrick H.J., LeBlanc M., Koo K., Askins C., Putnam M., Friebele E.J. (1997). Fiber grating sensors. J. Light. Technol..

[25] Weber M.J. (2002). Handbook of Optical Materials.

[26] Busch M., Ecke W., Latka I., Fischer D., Willsch R., Bartelt H. (2009). Inscription and characterization of Bragg gratings in single-crystal sapphire optical fibres for high-temperature sensor applications. Meas. Sci. Technol..

[27] Corless R.M., Gonnet G.H., Hare D.E.G., Jeffrey D.J., Knuth D.E. (1996). On the LambertW function. Adv. Comput. Math..

[28] Sakuma F., Kobayashi M. Interpolation equations of scales of radiation thermometers. Proceedings of the TEMPMEKO ’96, Sixth International Symposium on Temperature and Thermal Measurements in Industry and Science.

[29] Consultative Committee for Thermometry (2018). Guide to the Realization of the ITS-90–6—Radiation Thermometry.

[30] Barker D.G., Jones M.R. (2003). Inversion of spectral emission measurements to reconstruct the temperature profile along a blackbody optical fiber thermometer. Inverse Probl. Engng.

[31] (2013). IEC 60584-1:2013.

[32] Preston-Thomas H. (1990). The International Temperature Scale of 1990 (ITS-90). Metrologia.

[33] EURAMET TC-T (2020). Guidelines on the Calibration of Thermocouples.

[34] Burns G.W., Scroger M.G., Strouse G.F., Croarkin M.C., Guthrie W.F. (1993). Temperature-Electromotive Force Reference Functions and Tables for the Letter-Designated Thermocouple Types Based on the ITS-90.

[35] Reed R.P., Schooley J.F. (1992). Thermoelectric Inhomogeneity Testing: Part I—Principles. Temperature: Its Measurement and Control in Science and Industry.

[36] Webster E., White D.R. (2015). Thermocouple homogeneity scanning. Metrologia.

[37] Edler F., Huang K. (2020). Temperature Dependence of Thermoelectric Homogeneity of Noble Metal Thermocouples. Int. J. Thermophys..

